# Dietary supplementation with nano-zinc modulates the expression of the antiviral immune gene *IRF3*: a novel report

**DOI:** 10.3389/fimmu.2025.1583956

**Published:** 2025-08-04

**Authors:** Amrita Tah, Aruna Pal, Paresh Nath Chatterjee, Debasis De, Achinta Mahato, Argha Chakraborty, Manti Debnath, Sovan Roy

**Affiliations:** ^1^ Department of Fish Nutrition, West Bengal University of Animal and Fishery Sciences, Kolkata, India; ^2^ Department of Livestock Farm Complex, West Bengal University of Animal and Fishery Sciences, Kolkata, India; ^3^ Indian Council of Agriculture Research (ICAR)-Central Institute of Brakishwater Aquaculture, Kakdwip Research Centre, Kakdwip, India; ^4^ Department of Animal Nutrition, West Bengal University of Animal and Fishery Sciences, Kolkata, India; ^5^ West Bengal State Council of Science and Technology, Department of Science and Technology and Biotechnology, Kolkata, India

**Keywords:** nano-zinc, antiviral immunity, IRF3 expression, *Chanos chanos*, immune gene

## Abstract

In the era of severe acute respiratory syndrome coronavirus 2 (SARS-CoV-2), the application of zinc has increased worldwide. It has the potential to increase the body’s antioxidant status and provide better immune makeup. Currently, zinc is indiscriminately used as a therapeutic agent, and this creates an unwanted antagonism with the other interacting micronutrients that are present in the gut and causes secondary deficiencies of other critical micronutrients, which often leads to various complications. In this study, our aim was to synthesize a nano-sized zinc followed by its dietary fortification. We found that the supplemental nano-zinc has an antiviral effect using milkfish (*Chanos chanos*) as a model organism. The IRF3 gene was chosen as a molecular marker for antiviral assessment, which has different integral zinc binding sites. For the first time, we have characterized the IRF3 gene in *C. chanos* and discovered certain important domains as zinc binding sites, as well as other important domains related to antiviral activity, such as the serine protease NS3 activity and the interferon regulatory factor (IRF) tryptophan pentad repeat domain. The expression profile of IRF3 was significantly improved among fish supplemented with dietary nano-zinc, and the best effect was observed in the group provided feed fortified with 40 ppm nano-zinc. The results of this study revealed that nano-zinc can directly be incorporated into IRF3, which increases its bioavailability and improves its antiviral activity through biochemical pathways, as described in the STRING and Kyoto Encyclopedia of Genes and Genomes (KEGG) pathway analyses.

## Introduction

The recent advances in nanobiotechnology have reshaped scientific innovations with newer molecular insights to understand the basis of different biological processes. The higher surface-to-charge ratio offers nanoscale materials some unique properties to carry out desired functions. Research is in vogue to design different varieties of nanostructures for processing the production of vaccines, drugs, feed additives, nutrient delivery, disease treatment, and water purification, among others (1).

Minerals, as micronutrients, play a crucial role in the life processes of all categories of animals, including fish, and there is a need for their regular intake in order to combat physiological deficiencies. Merely fulfilling the requirements of a mineral might not assure its adequacy at the cellular level. The bioavailability of minerals appears to be the most challenging issue (2) in the science of nutrition. The bioavailability of a mineral is mostly regulated by its source and the presence of other interacting micronutrients in the gut. Recently, different varieties of nano-minerals have been synthesized, which appear to be highly bioavailable and hence have the least interaction with other nutrients available in the gut. Supplemental nano-minerals may have the potential to enhance growth and immunity (3). Synthesized nanoparticles appear to have more efficacy than their bulk counterparts in combating pathogens. The antimicrobial activity of several metal oxide nanoparticles has been proven against a wide range of bacteria and fungi (4).

Zinc is the second most abundant trace element in the body. However, it cannot be stored in the body, which emphasizes the need for regular dietary intake to avoid any subclinical deficiencies (5, 6). Zinc plays a vital role in prostaglandin metabolism and provides structural rigidity to nucleoproteins (7). It serves as an essential part of approximately 20 metalloenzymes, including alkaline phosphatase, alcohol dehydrogenase, and carbonic anhydrase, among others. In addition, zinc plays a critical role in growth performance, fertility, immune function, wound healing, oxidative stress maintenance, etc. (5, 8, 9). Zinc oxide and nano-zinc oxide have the same chemical structures, which suggests similar zinc and oxygen ratios. However, nano-sized atoms have a wider energy level confinement and, due to size variations, become less interactive with other bulk-sized molecules available in the gut (10). The higher bioavailability of nanostructures ensures adequacy at the tissue level; hence, nano-zinc appears to be more potent for immunomodulation in fish. It can increase the immunoglobulin M level and upregulate the expression of interleukin 1 beta (IL-1β (11). This higher immunomodulation may be associated with the increased thymocytes and peripheral T cells, enhancing the production of interferon and interleukin 2. Supplemental dietary zinc can improve the immune biomarkers that lead to a better immune response (12). Zinc oxide nanostructures appear potent immunomodulators even at lower doses compared with their bulk counterparts and organic zinc sources (11). Nano-zinc oxide can also promote the immune response by increasing the extra thymulin activity, which helps in the enhancement of mature T lymphocytes and the activation of B lymphocytes by T helper cells (12, 13). It has often been associated with increased lymphocyte count and phagocytic activity (11).

Interferon regulatory factors (IRFs) are transcription factors of the interferon (IFN) inducible signaling pathway and are crucial for host immunity against antimicrobial infection by both viruses and bacteria (14). IRFs are transcriptional regulators in the antiviral signaling pathway of the cell. When microbes attack the cell, IRFs bind to the IFN regulatory element (IRF-E) within the promoter region and regularize the transcriptional levels of the target genes. Thus, they play a pivotal role in the response to antiviral infections. There are different IRFs that play critical roles in innate immunity. IRF1, IRF3, IRF5, IRF7, IRF8, and IRF9 are among the positive regulators of IFN and other immune-related genes (15). IRF3 was found as a regulator of virus-infected cells 20 years ago (16). It promotes IFN-α/β gene transcription in virus-infected cells (17) and plays a very important role in controlling the IFN response during viral infection in mammals (18). IRF3 can also mediate apoptosis-independent p53 and type l IFN (19). There are nine members of IRF3 present in mammals (20), 10 members present in birds (21), and 11 members found in fish (18, 22). Studies on the innate immunity of fish are of recent interest considering the repeated outbreaks of pathogens in recent times. Several studies have reported that fish, similar to mammals, possess a defense mechanism in their innate immune system (14). Fish utilize the IFN-induced system against viral infections (14, 23). The induction of fish IFN through viral infection promotes antiviral activity and increases the interferon-stimulated gene (ISG) expression that reduces viral replication (14, 24). Several representative IRFs have been identified in fish, among which *Irf3* appears to play the key role in regulating the IFN response (14). Viral infection causes severe mortality in aquaculture, and it appears to cause great burden for farmers.

Attempts are in vogue to develop zinc-based feed additives with the aim of improving the body defense of fish. Being more bioavailable than and least antagonistic to other micronutrients, nano-zinc is presently gaining more attention. Fish are continuously being exposed to a plethora of stresses. The outbreak of various viral diseases is quite common in aquaculture and is a major issue for farmers. A number of sporadic experiments on dietary zinc for immunomodulation have been carried out in freshwater fish. However, there are scant reports available on brackish water fish. Milkfish (*Chanos chanos*) is rather delicious with an excellent nutrient profile and is thus quite popular in Southeast Asia and Taiwan (25). It is an important euryhaline finfish that can be cultured in freshwater, brackish water, or seawater (25, 26). In India, it has a high domestic market with good prices.

The present experiment aimed to compare the effect of formulated diets fortified with different sources of zinc (organic and nano-zinc) to evaluate its role in *IRF3* gene expression, which is essential for the antiviral defense of milkfish.

## Materials and methods

The present study aimed to determine the antiviral activity of nano-zinc in a milkfish (*C. chanos*) model. We synthesized the zinc nano-structures by employing an environment-benign colloidal chemistry route and characterized what has already been reported (27). They are suitable for mineral supplementation. IRF3 was employed as the molecular marker for assessment. We characterized the *IRF3* gene in *C. chanos* for the first time and assessed the differential messenger RNA (mRNA) expression level in the liver with respect to organic zinc and 10, 20, and 40 ppm nano-zinc.

### Experimental animals and dietary allocation

Apparently disease-free, healthy, motile *C. chanos* fries (0.35 ± 0.05 g) were procured from the pond of the Central Institute of Breakwater Aquaculture (CIBA), Kakdwip Research Centre, and were acclimatized for 15 days in 600-L fiber-reinforced plastic (FRP) tanks. The fish were fed twice daily (morning and evening) at 10% of their body weight. The waste was siphoned out and 80% water was exchanged at 3-day intervals to ensure the wellbeing of the fish with the least stress (28). No mortality was recorded during the present acclimatization period. The fish were observed for mortality daily, and dead fish were immediately removed. The water quality parameters (pH, dissolved oxygen, ammonia–nitrogen, etc.) were monitored regularly.

After the initial acclimatization, a total of 450 fish were randomly allocated into five treatment groups (**Table 1**), each with three replicates and each replicate containing 30 fish. The fish in T1 were fed the basal diet without any zinc supplementation; the fish in T2 were fed the basal diet supplemented with 20 ppm zinc proteinate; and the fish in the other three treatment groups (T3, T4, and T5) were supplemented with 10, 20, and 40 ppm nano-zinc oxide, respectively, as depicted in **Table 1**. The basal feeder remained isonitrogenous and isocaloric throughout the feeding trial across the treatment groups.

**Table 1:** Experimental design.

Group	Replicate	Dietary treatment
T1	3	Basal feed (without zinc)
T2	3	Basal feed + 20 ppm organic zinc
T3	3	Basal feed + 10 ppm nano-zinc
T4	3	Basal feed + 20 ppm nano-zinc
T5	3	Basal feed + 40 ppm nano-zinc

The feeding trial continued for 120 days and was followed by a digestibility trial extending for 5 days. At the end of the feeding trial, three fish from each replicate were collected in compliance with the guidelines for experimentation on fishes set by the Committee for the Purpose of Control and Supervision of Experiments on Animals (CPCSEA), Government of India. At the end of the feeding trial, three fish from each replicate were sacrificed by a registered veterinarian (PNC). The fish were firstly taken out from the tank and were euthanized using 0.1% of 100 ml clove oil at a rate of per liter of sterile water. The liver was stored in a sterile vial by keeping submerged in RNAlater solution (Invitrogen by Thermo Fisher Scientific, Waltham, MA, USA).

### Characterization of the *IRF3* gene in *C. chanos*

Total RNA was isolated from the liver using the TRIzol method and was subsequently converted to complementary DNA (cDNA) as per the standard protocol developed in our laboratory (29–32).

### Materials

Taq DNA polymerase, 10× buffer, and dNTP were purchased from Invitrogen (Carlsbad, CA, USA). The SYBR Green qPCR Master Mix (2×) was obtained from Thermo Fisher Scientific Inc. (Allentown, PA, USA). l-Glutamine (Glutamax 100×) was purchased from Invitrogen Corp., (Carlsbad, CA, USA), while penicillin-G and streptomycin were from Amresco (Solon, OH, USA). Filters (Millex GV., 0.22 µm) were purchased from Millipore Pvt. Ltd., (Billerica, MA, USA). All other reagents were of analytical and molecular biology grade.

### Synthesis, confirmation of cDNA, and PCR amplification of the gene

The 20-μl reaction mixture contained 5 μg of total RNA, 0.5 μg of oligo dT primer (16–18 mer), 40 U of ribonuclease inhibitor, 10 M of dNTP mix, 10 mM of DTT, and 5 U of MuMLV reverse transcriptase in reverse transcriptase buffer. The reaction mixture was gently mixed and incubated at 37°C for 1 h. The reaction was stopped by heating the mixture at 70°C for 10 min and chilling on ice. The integrity of the cDNA was assessed by PCR. The primers used are listed in **Tables 2**, **3**. The 25-μl reaction mixture contained 80–100 ng cDNA, 3.0 μl 10× PCR assay buffer, 0.5 μl of 10 mM dNTP, 1 U Taq DNA polymerase, 60 ng of each primer (as in **Table 2**), and 2 mM MgCl_2_. The PCR reactions were carried out in a thermocycler (PTC-200; MJ Research, Hercules, CA, USA), with cycling conditions as follows: initial denaturation at 94°C for 3 min, denaturation at 94°C for 30 s, annealing temperature of 60°C for 35 s, and extension at 72°C for 3 min carried out for 35 cycles, followed by a final extension at 72°C for 10 min.

**Table 2:** Primers for the amplification of the *IRF3* gene in *Chanos chanos*.

Fragment	Primer sequence	
1	Forward	CCGTTACATCAGCCGGAATCT
	Reverse	GAGGAGTTTTCACCAGGCCA
2	Forward	GGACCAACACAAACTCAGCC
	Reverse	GACCCTCTACCCTGACCTCC
3	Forward	CACTGGGTATTGGGCCCTTT
	Reverse	ACCTGTCACGTAATCTCTGGC
4.	Forward	CCTGAACCCATCTACTCAGCC
	Reverse	ACAGAGAACATTGTGGCATTCA
5	Forward	TGTTATAGAACATCGTACAGTGAATGT
	Reverse	CCTAAAGGGCCAATGATGTAGA

### Study of the predicted gene using bioinformatics tools

The predicted peptide sequences of the genes of milkfish were derived using Edit sequence (Lasergene software; DNASTAR, Madison, WI, USA). Prediction of the signal peptide of the genes was conducted using the software SignalP3.0 Server—prediction results (Technical University of Denmark). Analysis of the *O*-linked glycosylation sites was carried out using the NetOGlyc 4 server (https://www.expassy.org/), whereas the *N*-linked glycosylation sites were detected using NetNGlyc 1.0 software (https://www.expassy.org/). The regions for the alpha-helix and beta-sheet were predicted using NetSurfP-Protein Surface Accessibility and Secondary Structure Predictions, Technical University of Denmark (33). Domain linker prediction was performed according to the software developed (34). The lipopolysaccharide (LPS) binding sites (35), as well as the LPS signaling sites (36), were predicted based on homology studies with other species of polypeptide.

### Three-dimensional structure prediction and model quality assessment

The templates with the highest sequence ID entity to our target template were identified using PSI-BLAST (http://blast.ncbi.nlm.nih.gov/Blast). Homology modeling was used to build a three-dimensional (3D) structure based on homologous template structures using the PHYRE2 server (37). The 3D structures were visualized with PyMOL (http://www.pymol.org/), which is an open-source molecular visualization tool. Subsequently, the mutant model was generated using PyMoL tools. The Swiss PDB Viewer was employed to control energy minimization. Structural evaluation, along with a stereochemical quality assessment of the predicted model, was carried out using SAVES (Structural Analysis and Verification Server), which is an integrated server (http://nihserver.mbi.ucla.edu/SAVES/). The ProSA (Protein Structure Analysis) web server (https://prosa.services.came.sbg.ac.at/prosa) was used for the refinement and validation of the protein structure (38). ProSA was used to evaluate the structural quality of the model with potential errors, and the program shows a plot of its residue energies and *Z*-scores, which determine the overall quality of the model. The solvent accessibility surface area of the genes was generated using the NetSurfP server (https://www.cbs.dtu.dk/services/NetSurfP/) (33). This calculates the relative surface accessibility, the *Z*-fit score, the probabilities for both the alpha-helix and the beta-strand, and the coil score, among others. TM-align software was used for the alignment of the 3D structure of the IR proteins for different species and for the estimation of the root mean square deviation (RMSD) to assess structural differentiation (39). I-Mutant analysis was conducted for the mutations detected to assess the thermodynamic stability. PROVEAN analysis was conducted to assess the deleterious nature of the mutant amino acid. The PDB structure for the 3D structural prediction of the gene for milkfish was carried out using PHYRE software (38). Protein–protein interactions were examined using STRING analysis (40).

### Differential mRNA expression profiling of the *IRF3* gene in *C. chanos*

#### Real-time PCR

Total RNA was estimated from the liver of milkfish from the six treatment groups using the TRIzol method. First-strand cDNA was synthesized using reverse transcriptase polymerase chain reaction (RT-PCR) in an automated temperature-maintained thermocycler machine. M-MLVRT (200 U/µl) was used as the reverse transcriptase enzyme. The primer was obtained from a published journal (25). The primers used are listed in **Table 3**. Equal amounts of RNA (quantified with a Qubit fluorometer; Invitrogen, Carlsbad, CA, USA), whenever applicable, were used for cDNA preparation (SuperScript III cDNA Synthesis Kit; Invitrogen, Carlsbad, CA, USA). All qRT-PCR reactions were conducted on an ABI 7500 Fast system. Each reaction consisted of a 2-µl cDNA template, 5 µl of 2× SYBR Green PCR Master Mix, 0.25 µl each of forward and reverse primers (10 pmol/µl), and nuclease-free water for a final volume of 10 µl. Each sample was run in triplicate. Analysis of real-time PCR (qRT-PCR) was performed using the delta–delta *C*_t_ (ΔΔ*C*_t_) method (29, 30, 32, 41).

**Table 3:** PCR primers for differential mRNA expression profiling of the *IRF3* gene and the housekeeping gene in *Chanos chanos* (25).

Gene	Primer sequence	
*IRF3*	Forward	TCC TTG GGT TTA TGC ACA CC
	Reverse	ATT CCC TCA GAC CTG TCA CG
*Beta-actin*	Forward	GACGGACAGGTCATCACCATTGGC
	Reverse	GGTGTTGGCGTACAGGTCCTTACG

The entire reactions were performed in triplicate (as per the MIQE guidelines), and the experiment was repeated twice, in a 20-µl reaction volume, using FastStart Essential DNA Green Master (HiMedia, Mumbai, India) on the ABI 7500 system.

### Statistical analysis

Descriptive statistics with the mean and standard error were estimated with the SYSTAT package for the expression levels analyzed using real-time PCR and accordingly presented in a graph. The expression level with real-time PCR was estimated using 2^−ΔΔCt^.

## Results

### Characterization of the *IRF3* gene in *C. chanos*

#### Characterization of the *IRF3* gene: *in silico* studies and identification of the important domains

**Figure 1:**
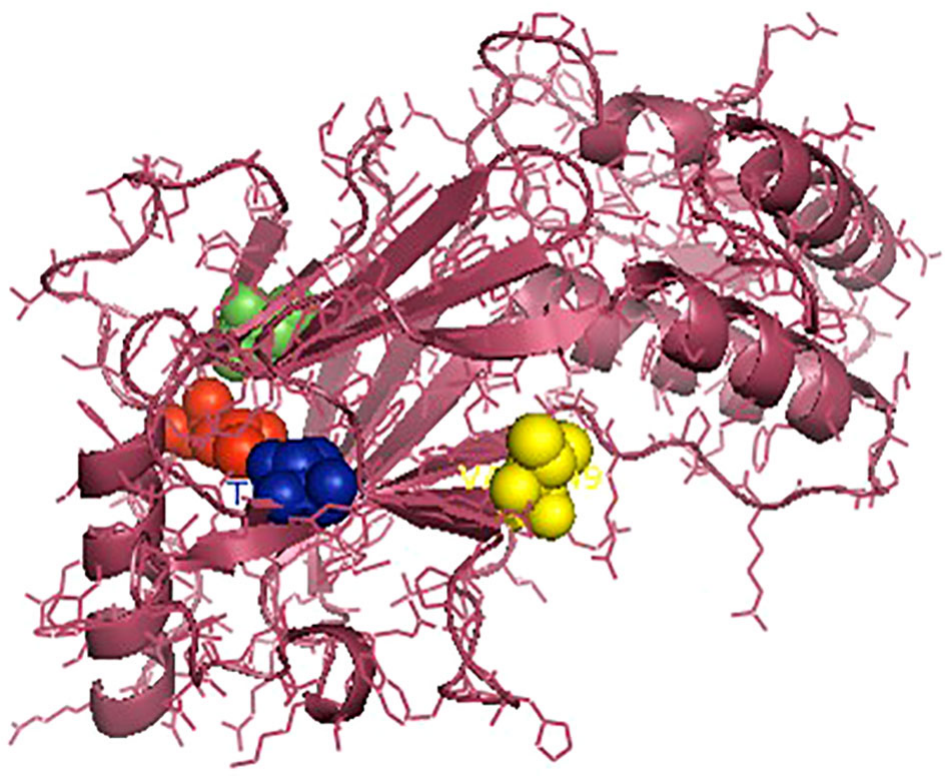
Three-dimensional structure of *IRF3* in *Chanos chanos* with the domains for the zinc binding sites. Zinc binding sites: amino acid (aa) 97 (*green* sp*here*); aa 99 (*red* sp*here*); aa 145 (*blue* sp*here*); and aa 149 (*yellow* sp*here*).

**Figure 2:**
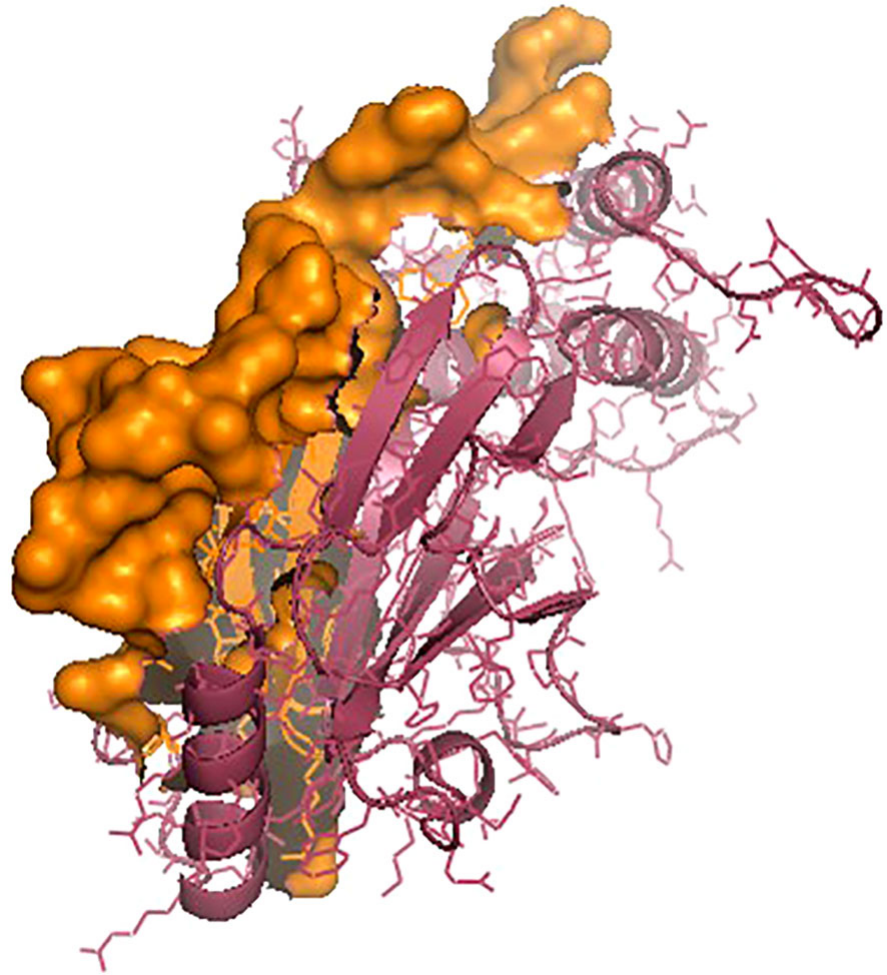
IRF3 protein with the interferon regulatory factor (IRF) tryptophan pentad repeat domain (amino acids 5–102) as the *orange surface*.

**Figure 3:**
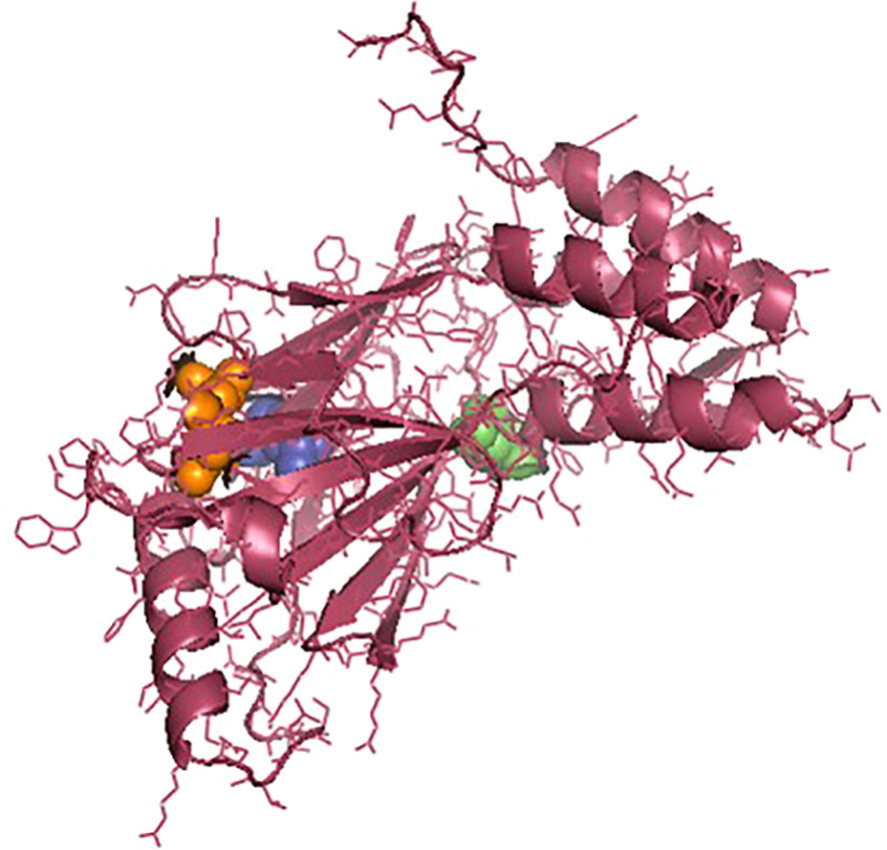
Three-dimensional structure of *IRF3* in *Chanos chanos* with the domains for serine protease NS3 activity. Amino acid (aa) 57 (*green sphere*); aa 81 (*blue* sp*here*); and aa 139 (*orange* sp*here*).

**Figure 4:**
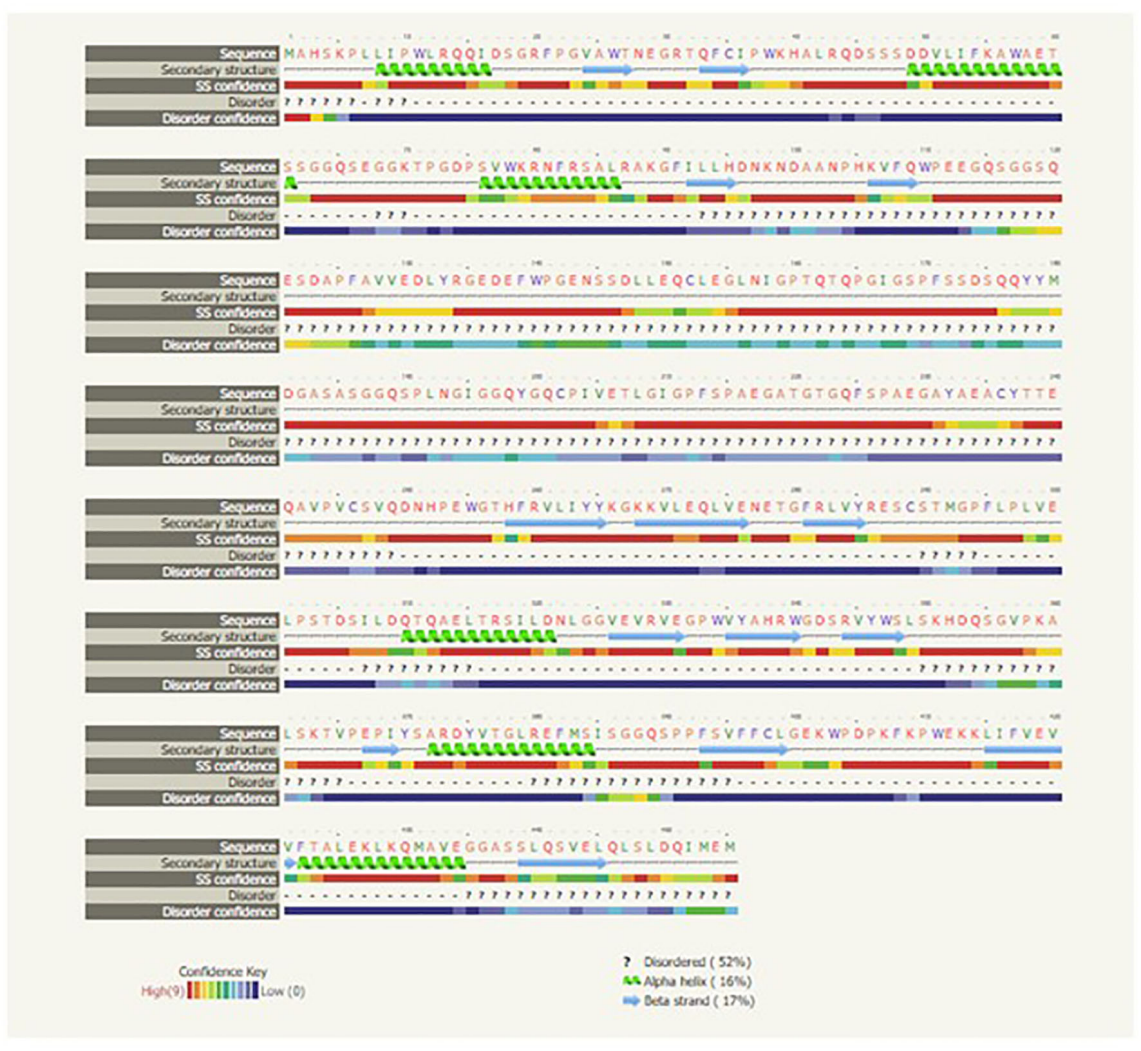
Secondary structure of the *IRF3* gene for *Chanos chanos*.

The *IRF3* gene has been employed in current studies as an important molecular marker against viral infections. The *IRF3* gene has been characterized and the predicted 3D structure for the peptide sequence visualized (**Figure 1**), with a predicted interferon regulatory factor (IRF) tryptophan pentad repeat domain at amino acid positions 5–102 (**Figure 2**, orange surface). The serine protease NS3 domain is also depicted in **Figure 3**. The zinc binding sites were detected at amino acid position 97 (**Figure 1**, green sphere), position 99 (**Figure 1**, red sphere), position 145 (**Figure 1**, blue sphere), and position 149 (**Figure 1**, yellow sphere). The secondary structure of the *IRF3* gene of *C. chanos* is depicted in **Figure 4**.

### STRING analysis

**Figure 5:**
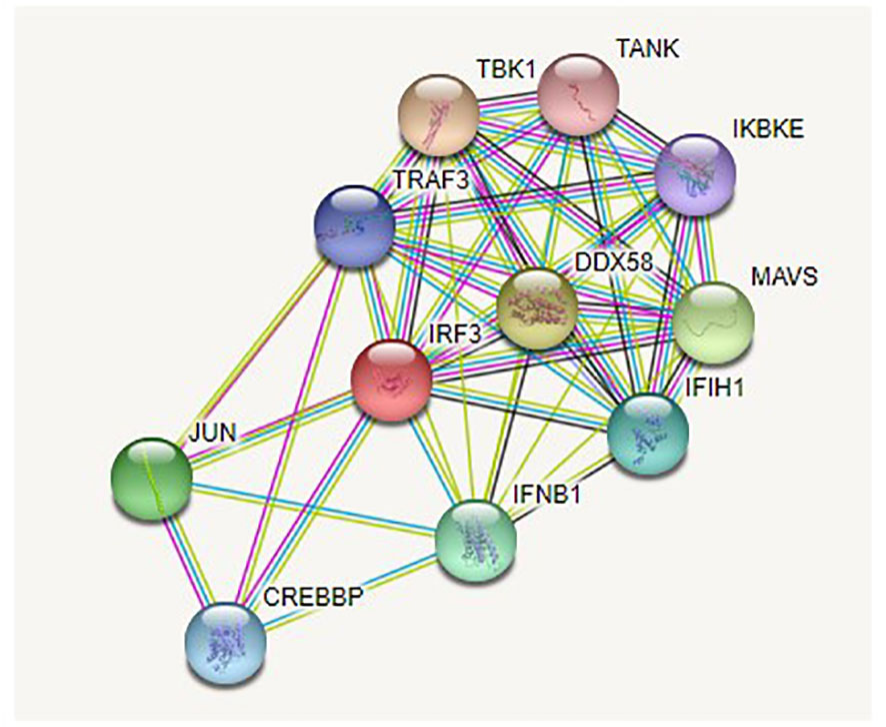
STRING analysis of the *IRF3* gene for *Chanos chanos*.

**Figure 6:**
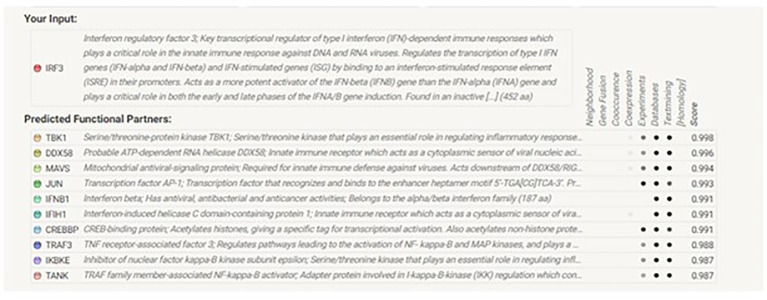
Genes interacting with *IRF3* in *Chanos chanos*.

The analysis revealed that *IRF3* interacts with many other genes in a complex biological process. According to their interaction scores, these genes included *DDX58*, *MAVS*, *IFNB1*, *IFIH1*, *TRAF3*, *TBK1*, *TANK*, *IKBKE*, *JUN*, and *CREBBP* (**Figures 5**, **6**).

### KEGG analysis

**Figure 7:**
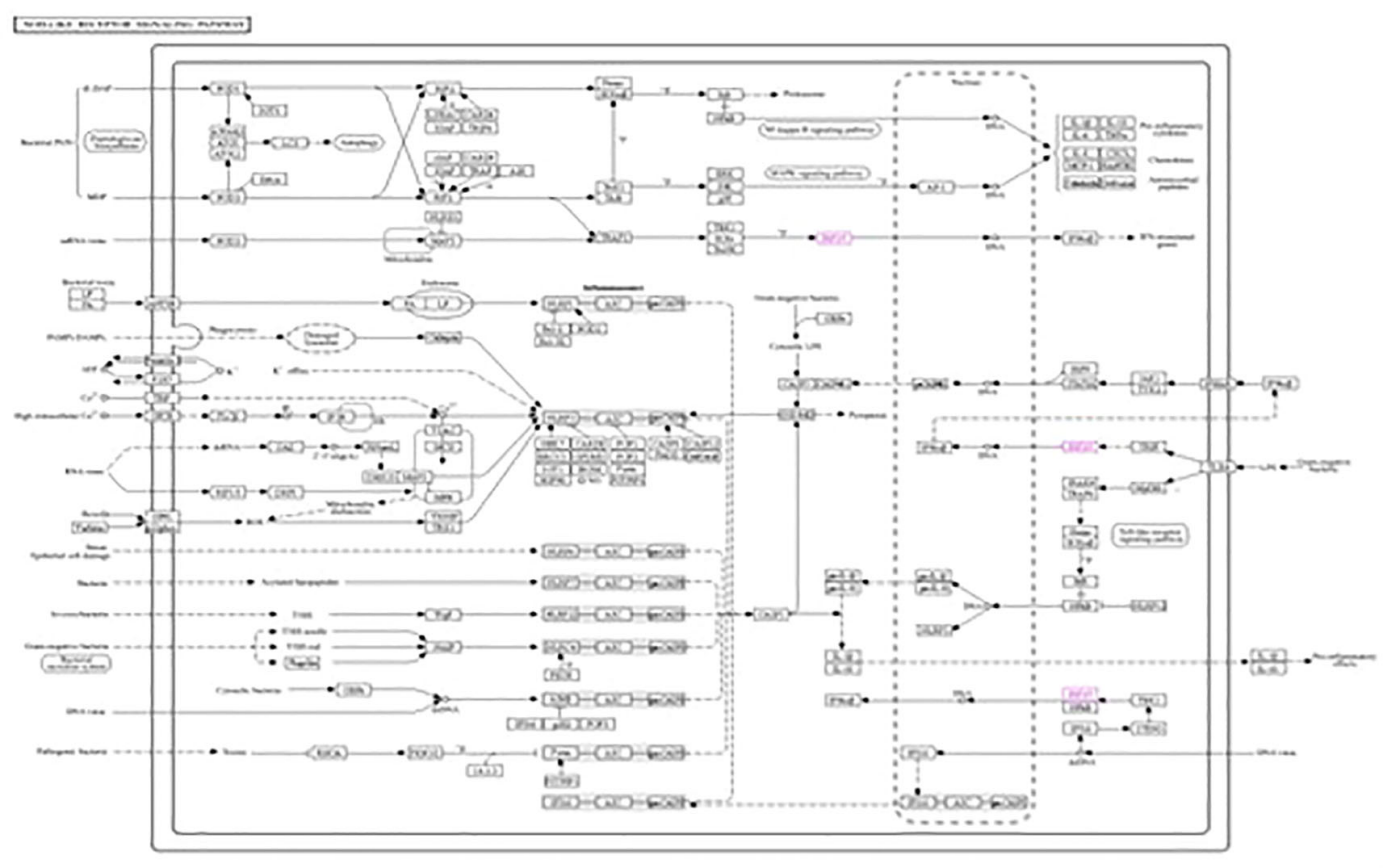
Kyoto Encyclopedia of Genes and Genomes (KEGG1) analysis of *IRF3* showing the antiviral signaling pathway through the NOD-like receptor pathway.

**Figure 8:**
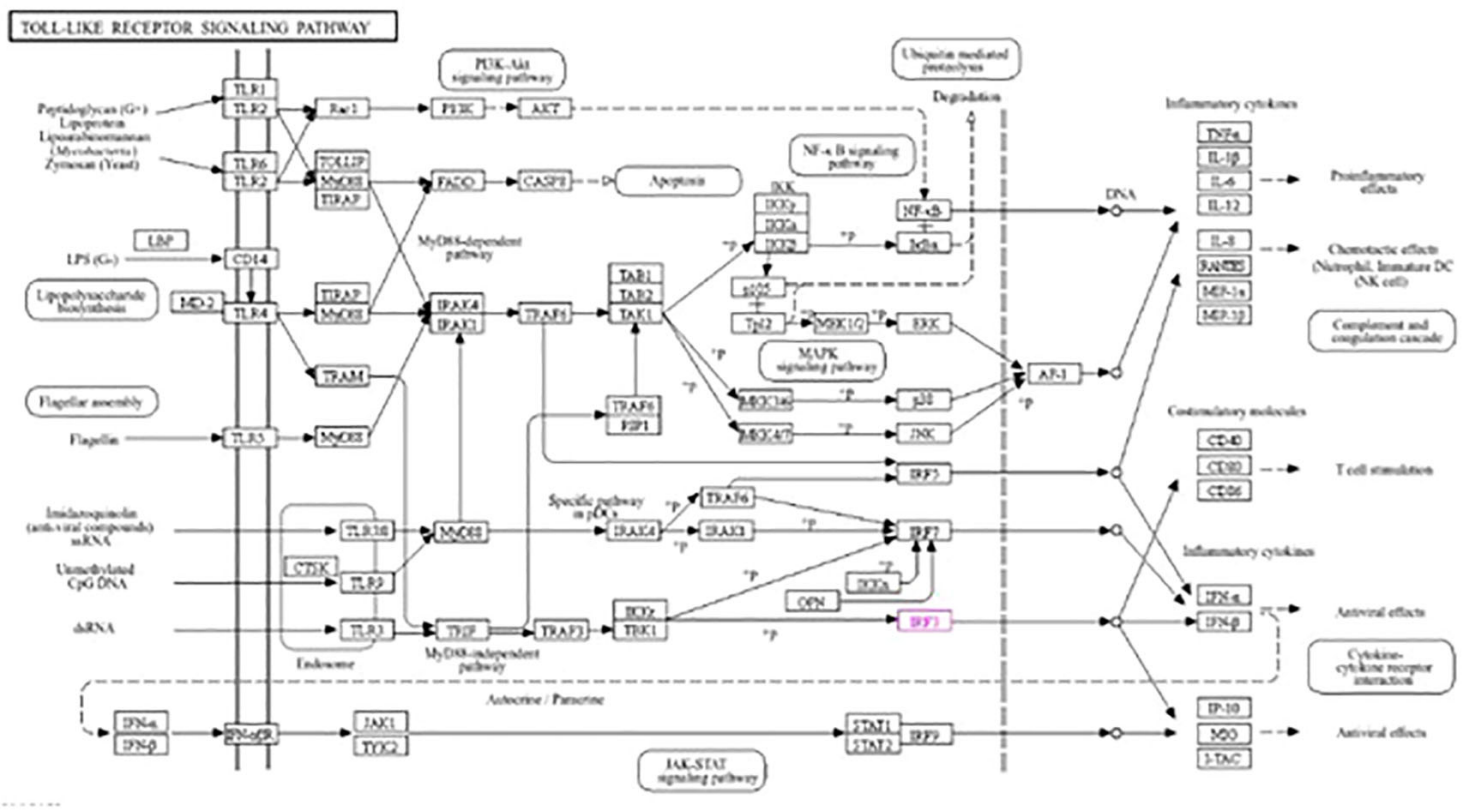
Kyoto Encyclopedia of Genes and Genomes (KEGG2) analysis of *IRF3* showing the antiviral signaling pathway through the Toll-like receptor pathway.

**Figure 9:**
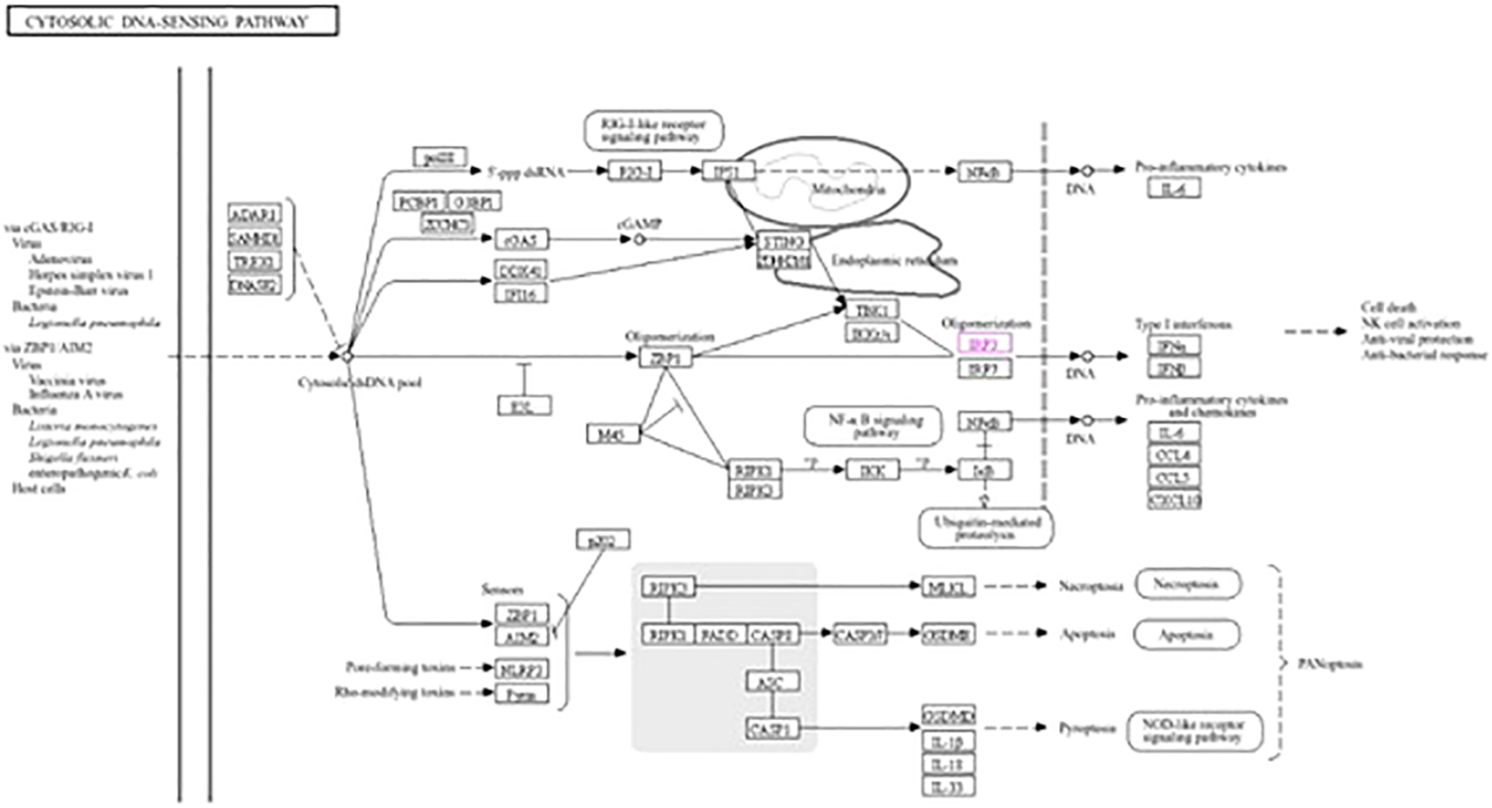
Kyoto Encyclopedia of Genes and Genomes (KEGG3) analysis of *IRF3* showing the antiviral signaling pathway through the cytosolic DNA sensing pathway.

The roles of *IRF3* in NOD-like receptor signaling, the Toll-like receptor signaling pathway, and the cytosolic DNA sensing pathway are described in **Figures 7**–**9**, respectively. The analysis also provided proof of the antiviral effect of *IRF3* through these aforementioned pathways.

### Differential mRNA expression profiling of the gene

**Figure 10:**
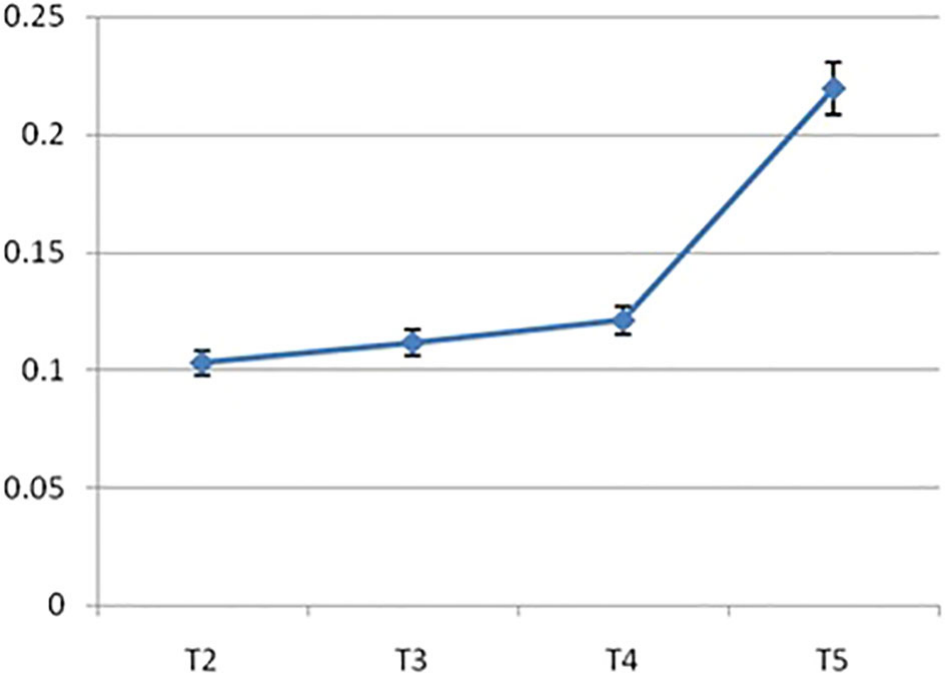
Differential mRNA Expression profiling of IRF3 gene of Chanos chanos upon feeding with zinc of different sources. (T2: Organic zinc, T3: Nano zinc 10ppm, T4: Nano zinc 20ppm, T5: Nano zinc 40 ppm).

The *IRF3* gene was observed to be upregulated in group T5 (40 ppm nano-zinc-supplemented group) compared with that of the other treatment groups (**Figure 10**). The lowest expression was observed in the organic zinc-supplemented treatment group, i.e., group T2 (20 ppm zinc supplementation). However, the 10- and 20-ppm nano-zinc-supplemented groups showed better expression of *IRF3* than the T2 group. When the concentration of nano-zinc increased to 40 ppm, the expression of the *IRF3* gene was also highly upregulated. The expression of the *IRF3* gene gradually increased in three nano-zinc supplement treatments: T3 (10 ppm nano-zinc supplementation), T4 (20 ppm nano-zinc supplementation), and T5 (40 ppm nano-zinc supplementation).

## Discussion

Zinc is an extremely critical micronutrient needed in an adequate amount at the tissue level in order to impart proper antioxidant status and optimum immunity to fight against pathogens. The antibacterial effects of nano-zinc have already been established (27, 42, 43). The bioavailability of zinc has been recorded to be quite high and with good antimicrobial potential (27). For dietary fortification, synthesized nano-ZnO was used at up to 40 ppm based on the recommendation of our earlier studies (27, 44).

To overcome the issues of bioavailability and to impart a higher antioxidant status, attempts are in vogue (27, 45, 46) to develop different varieties of nano-zinc for use in dietary fortification. A number of researchers have synthesized a zinc nanoparticle (206 nm) and observed its positive impact on the immune response and antioxidant status of fish (47). Different varieties of zinc nanoparticles (size range between 105.7 and 122.4 nm) have also been devised and evaluated for their efficacy in different fish models (48). One special variety of dietary nano-zinc oxide was synthesized with a particle size of 50–60 nm and fortified the diet of grass carp with the aim of imparting better body defense (49). The particle sizes of the synthesized nano-zinc ranged from 10 to 50 nm and from 20 to 120 nm, as confirmed by transmission electron microscopy (TEM) and dynamic light scattering (DLS) studies (50).

Zinc has already been reported to possess an antiviral effect against retroviruses, such as severe acute respiratory syndrome coronavirus (SARS-CoV) and SARS-CoV-2, among others (51). Several studies have proven that zinc has antiviral activity against hepatitis virus (52). Zinc oxide nanoparticles protected the Madin–Darby bovine kidney (MDBK) cell culture and experimental rabbits that were infected with herpesvirus (53). It also has the potential to induce varied gene expression related to immunity, including Toll-like receptors, tumor necrosis factor (TNF), and IL-1β, among others (54). However, research has been limited at a certain level, and there is no report elucidating the molecular mechanism.

We found several reports demonstrating the effect of nano-zinc on viruses and the mechanism of how they destroy a particular virus. However, our aim was to determine the molecular mechanism and the effect of synthesized nano-zinc on the expression of a gene that initiates signaling against viral infection through studying the detailed molecular structure using bioinformatics tools. This experiment attempted to assess the antiviral potential of nano-zinc, with *IRF3* as a marker gene. *IRF3* is considered to be one of the most important factors in antiviral signaling (15). In aquaculture, virus infection is one of the major threats (55), which often causes serious economic losses (56). When fishes are infected with a virus, severe mortality occurs (57), along with associated morbidity losses. It is a challenge to fight viral diseases in aquaculture. Therefore, there is an urgent need to search for a natural ingredient/additive to provide a considerable degree of protection against sudden viral infections.

This study showed that nano-zinc upregulates the expression of the *IRF3* gene. *IRF3* can fight against both RNA and DNA viruses (58, 59). Kyoto Encyclopedia of Genes and Genomes (KEGG) analysis showed that *IRF3* is involved in three antiviral signaling pathways: the cytosolic DNA sensing pathway, the NOD-like receptor signaling pathway, and the Toll-like receptor signaling pathway. When an RNA virus infects the cell, the NOD-like receptor signaling pathway is activated in the cytosol by the activation of *IRF3* through *MAVS* and *TRAF3* (60, 61). This activated *IRF3* then stimulates the activation of IFNαβ and initiates antiviral activity. When the double-stranded DNA is recognized by TLR3 in the endosome in the Toll-like receptor pathway, *IRF3* is stimulated through *TRIF*, *TRAF3*, and *TBK1* by interacting with *TLR3* (62). Subsequently, IFNα and IFNβ are activated by *IRF3* and initiate an antiviral signaling pathway. In the case of the cytosolic DNA binding pathway, when the cell is infected with a virus, *IRF3* is activated through *ZBP1*, *TBK1*, and *IKK*. Now activated, *IRF3* then stimulates the production of IFNα and IFNβ and provides cell antiviral protection (58, 63). *IRF3* initiates antiviral activity by activating two factors, i.e., IFNα and IFNβ (14, 15).

*IRF3* has a tryptophan pentad domain that helps in the cellular response to viruses. This domain can recognize any change in the state or activity of a cell that is mediated by the stimulus of a virus (https://www.ebi.ac.uk/QuickGO/term/GO:0098586). There is another domain present in *IRF3*, i.e., the NS3 domain. It plays an important role in viral infection. NS3 hydrolyzes the four peptides bonded in the viral precursor polyprotein; thus, NS3 can destroy the viruses that infect the host cell (https://ftp.expasy.org/databases/prosite). The activated *IRF3* functions against viral pathogens through the tryptophan pentad domain and the NS3 domain.

Organic zinc and nano-zinc differ in particle size. The smaller size of nano-zinc provides a high surface area, bioavailability, and more efficacy than organic zinc. Organic zinc is always in competition with other micronutrients and cannot be properly absorbed. Due to this, bulk-sized zinc cannot easily enter the IRF3 protein and cannot be placed properly through all the different zinc binding sites of the protein. This is why organic zinc fails to upregulate the expression of the *IRF3* gene. On the other hand, nano-zinc is small and there is no chance to compete with other micronutrients. It can also easily enter the protein and properly bind with the different zinc binding sites of the protein. When nano-zinc binds with the IRF3 protein, it activates *IRF3* against viral pathogens by activating the tryptophan pentad domain and the NS3 domain and stimulating its expression. When the concentration of nano-zinc increases, more zinc particles can accumulate with the protein and increase its expression.

From this experiment, it can be revealed that nano-zinc stimulates the signaling against viral pathogens by upregulating the IRF3 protein expression, and *IRF3* stimulates IFNα and IFNβ, which then initiate antiviral signaling. Nano-zinc has the potential to fight against viral infection in the cell.

## Conclusion

The *IRF3* gene plays an extremely crucial role in the antiviral pathway. In the present study, the highest expression was recorded among fish that received feed fortified with 40 ppm nano-zinc. The corresponding STRING and KEGG pathway analyses revealed the possible mechanism of the higher *IRF3* expression as induced by nano-zinc. Due to the higher bioavailability of nano-zinc, it can saturate the zinc binding sites present in *IRF3* and can modulate its expression profiling in *C. chanos.* The present study explored the possible mechanism of action of nano-zinc in conferring antiviral immunity via the upregulation of the *IRF3* gene, which could be used as a molecular marker to assess the antiviral protection in *C. chanos.* Nano-zinc oxide not only acts as an antibacterial agent but can also be used as a potential antiviral agent.

### Future scope

In the future, this study could be propagated with challenging studies against pathogenic viruses by maintaining an appropriate biosecurity protocol.

## Data Availability

The datasets presented in this study can be found in online repositories. The names of the repository/repositories and accession number(s) can be found in the article/supplementary material.
